# COVID-19 Reinfections in the City of São Paulo, Brazil: Prevalence and Socioeconomic Factors

**DOI:** 10.1093/ofid/ofaf181

**Published:** 2025-04-16

**Authors:** Daniel Tavares Malheiro, Kauê Capellato Junqueira Parreira, Patricia Deffune Celeghini, Gustavo Yano Callado, André Luis Franco Cotia, Miguel Cendoroglo Neto, Marcelo A S Bragatte, Isaac Negretto Schrarstzhaupt, Vanderson Sampaio, Takaaki Kobayashi, Michael B Edmond, Alexandre R Marra

**Affiliations:** Hospital Israelita Albert Einstein, São Paulo, Brazil; Hospital Israelita Albert Einstein, São Paulo, Brazil; Hospital Israelita Albert Einstein, São Paulo, Brazil; Hospital Israelita Albert Einstein, São Paulo, Brazil; Hospital Israelita Albert Einstein, São Paulo, Brazil; Hospital Israelita Albert Einstein, São Paulo, Brazil; Instituto Todos Pela Saúde, São Paulo, Brazil; Instituto Capixaba de Ensino, Pesquisa e Inovação em Saúde ICEPi, Espírito Santo, Brazil; Instituto Todos Pela Saúde, São Paulo, Brazil; Instituto Capixaba de Ensino, Pesquisa e Inovação em Saúde ICEPi, Espírito Santo, Brazil; Faculdade de Saúde Pública, Universidade de São Paulo, São Paulo, Brazil; Instituto Todos Pela Saúde, São Paulo, Brazil; Department of Internal Medicine, University of Kentucky, Lexington, Kentucky, USA; Department of Internal Medicine, University of Iowa, Iowa City, Iowa, USA; West Virginia University School of Medicine, Morgantown, West Virginia, USA; Hospital Israelita Albert Einstein, São Paulo, Brazil; Department of Internal Medicine, University of Iowa, Iowa City, Iowa, USA

**Keywords:** Brazil, COVID-19 reinfection, prevalence, SARS-CoV-2 variants, health disparity

## Abstract

**Background:**

Identifying those most susceptible to COVID-19 reinfection and understanding the associated characteristics is essential for developing effective prevention and control strategies. We aimed to evaluate the influence of social determinants, regional disparities, and variant evolution on COVID-19 reinfection rates.

**Methods:**

We conducted a retrospective cohort study in São Paulo, Brazil, involving laboratory-confirmed COVID-19 patients. Reinfection was defined as a subsequent positive COVID-19 test at least 90 days after the previous confirmed infection. We assessed socioeconomic indicators, demographic factors, and spatial correlations. Reinfection rates were analyzed across different variants and subvariants.

**Results:**

Among 73 741 patients, 5626 (7.6%) experienced reinfections, with most (95.0%) having 1 reinfection. Reinfection rates increased significantly during the Omicron period, particularly with subvariants BA.1, BA.2/BA.4, BA.5, and XBB/XBB.1.5/XBB.1.16. The highest rates were seen in patients initially infected during the BA.2/BA.4 and BA.5 periods, who were later reinfected by XBB subvariants. Socioeconomic indicators, including lower Human Development Index, higher proportions of informal settlements, and lower employment rates, were significantly associated with higher reinfection rates. Geospatial analysis showed significant clustering of reinfections in areas with higher social vulnerability.

**Conclusions:**

COVID-19 reinfection rates were heavily influenced by socioeconomic disparities and variant-specific factors. Regions with lower Human Development Index and worse socioeconomic conditions experienced higher reinfection rates. These findings highlight the need for targeted public health interventions focused on vulnerable populations, particularly in areas with greater social inequality. As new variants continue to emerge, ongoing surveillance and adaptive public health strategies will be critical to reducing reinfections.

## Teaser

COVID-19 reinfection rates in São Paulo were driven by socioeconomic disparities and variant evolution, with higher rates in socially vulnerable areas. Findings support targeted interventions and adaptive strategies to protect high-risk populations.

## Graphical Abstract

**Graphical Abstract:**
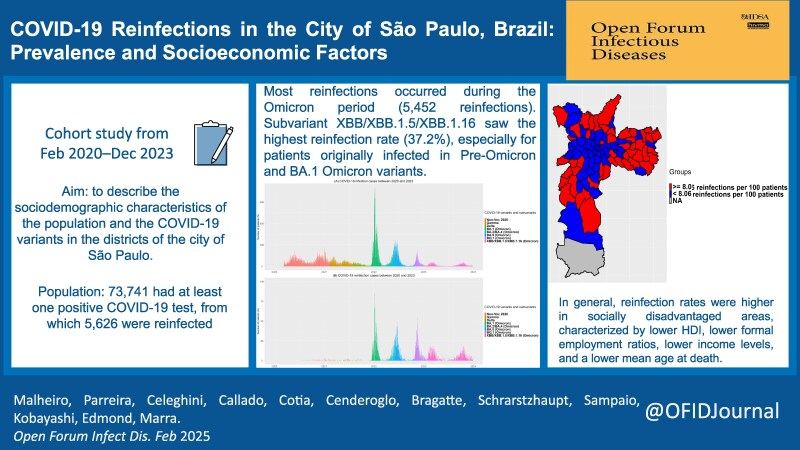

## BACKGROUND

COVID-19 reinfection remains a significant public health issue, despite broad vaccine coverage and prior infections. Understanding the characteristics of those most at risk is essential for developing targeted prevention and control strategies [1]. The emergence of the Omicron variant in late 2021 reshaped reinfection dynamics, with Omicron's mutations enabling it to bypass existing immunity, resulting in more frequent reinfections, even among vaccinated or previously infected individuals [2].

Social disparities significantly influence the risk and impact of COVID-19 infections [3]. Populations in lower socioeconomic groups often face healthcare barriers, live in crowded conditions that facilitate viral spread, and work in jobs that lack remote options, thereby increasing exposure risk. These factors contribute to higher infection rates among disadvantaged groups, underscoring the need for public health interventions addressing these inequalities [4]. Individuals with lower socioeconomic status are more likely to live in multigenerational households or work in essential services, environments that elevate exposure risk [5]. Additionally, individuals with socioeconomic disadvantages may face barriers such as lack of health insurance, high medical costs, transportation difficulties, and limited availability of healthcare providers [6].

Identifying risk factors for reinfection supports public health officials in targeting interventions and resource allocation toward high-risk groups and enables individuals to take informed health precautions. Research on these risk factors also expands understanding of the virus, potentially improving prevention and treatment strategies [7]. Given Brazil's high infection burden, significant social inequalities, and São Paulo's status as the most populous state, understanding reinfection dynamics in this region is crucial for developing effective public health responses [8].

This study aims to describe the sociodemographic characteristics of populations experiencing higher reinfection rates and the COVID-19 variants involved, using a database of laboratory-confirmed COVID-19 individuals in São Paulo, Brazil. The study also seeks to examine the impact of emerging variants and subvariants on reinfection rates, with a focus on social vulnerability and health disparities.

## METHODS

### Study Design, Location and Participants

This was a single-center, retrospective cohort study, which included all symptomatic patients with a confirmed diagnosis of COVID-19 at Hospital Israelita Albert Einstein (HIAE) from 24 February 2020, to 31 December 2023. HIAE is a nonprofit healthcare, educational, and research organization, with headquarters in the city of São Paulo, managing diverse services from primary to tertiary care, in the public and private healthcare sectors. It operates 40 healthcare units, mainly in the state of São Paulo, with a centralized laboratory that process tests from all these units, including hospitalized patients, emergency room visits, and outpatient services.

All individuals with a laboratory-confirmed COVID-19 were included. Laboratory confirmation was performed using real-time polymerase chain reaction on specimens obtained via nasopharyngeal swab. Additionally, we considered the antigenic test and the detection of COVID by metagenomic next-generation sequencing as valid laboratory confirmation tests for COVID-19. The study was approved by the Ethics Committee of HIAE—CEP/Einstein.

### COVID-19 Reinfection Definition

COVID-19 reinfection was defined as a subsequent positive COVID-19 test occurring at least 90 days after the last confirmed infection [9].

### Data Collection and Measures

Data were collected using an electronic medical record. For each patient, admission information such as age, sex, the neighborhood of residence of the individual and positive COVID-19 test date(s) were collected. Socioeconomic indicators were obtained from the “São Paulo's city inequality map,” which is a tool published by a social society organization “Rede Nossa Vida.” This map compiles data from various areas of municipal public administration, disaggregated by district, to allow comparisons and identify regions most lacking in services and urban infrastructure. The tool aims to assist public managers in identifying priorities and addressing the specific needs of each district within the capital [10]. Detailed descriptions of each indicator are provided in Supplementary Material 1. Additionally, the Human Development Index (HDI) for each district of São Paulo was obtained from the study by Brito et al [11]. The HDI is calculated as a geometric mean of 3 dimensions: life expectancy, education, and living standards. It ranges from 0 to 1, where 0–0.499 reflects “very low human development,” 0.5–0.599 indicates “low human development,” 0.6–0.699 represents “medium human development,”’ 0.7–0.799 means “high human development,” and 0.8–1 indicates “very high human development” [12]. Favelas are informal urban settlements, often characterized by high population density, limited infrastructure, and a lack of formal housing regulation, which contributes to socioeconomic disparities within the districts. Air pollution refers to the amount of pollutants emitted annually (kg/year) within a district, adjusted per square kilometer (km^2^) of geographic area.

The primary outcome variable in this study was “COVID-19 reinfection rate”, which was calculated as the number of first COVID-19 reinfections divided by the total number of unique patients, expressed per 100 patients. The median reinfection rate was calculated as the middle value of reinfection rates across all districts, where 50% had rates equal to or higher. Visual maps were plotted using São Paulo's shapefile for the 96 districts of the city, provided by the Municipal Department of Urban Planning and Licensing of the City Hall of São Paulo [13].

### Period of COVID-19 Variants

**Figure 1.**
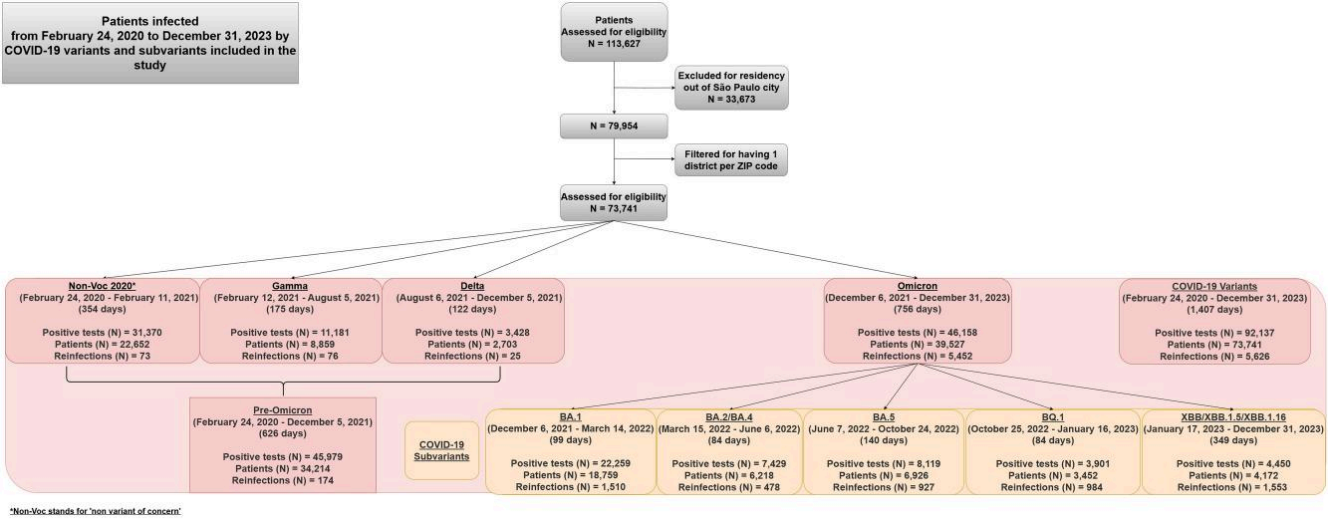
Patients with COVID-19 infection and reinfection from 24 February 2020 to 31 December 2023 by variants and subvariants included in the study.

Because only a small number of positive samples among our cases were sequenced, all individuals were classified according to the most prevalent variant. Classification was based on the predominant variant (≥75% of sequenced viruses) reported weekly in São Paulo, Brazil, by the Global Initiative on Sharing Avian Influenza Data [14]. In summary, the period from 24 February 2020 to 11 February 2021 was considered the “non–variant-of-concern 2020 (Non-Voc 2020)”, the period 12 February 2021 to 5 August 2021 was identified as the “Gamma” variant period, followed by the “Delta” variant period from 6 August 2021 to 5 December 2021. The “Omicron” variant period extended from 6 December 2021 to 31 December 2023 and was further divided into 5 subvariants: BA.1 from 6 December 2021 to 14 March 2022; BA.2/BA.4 from 15 March 2022 to 6 June 2022; BA.5 from 7 June 2022 to 24 October 2022; BQ.1 from 25 October 2022 to 16 January 2023; and XBB/XBB.1.5/XBB.1.16 from 17 January 2023 to 31 December 2023 [Figure 1].

### Statistical Analysis

To compare the demographic characteristics of patients who did not experience reinfection with those who did, Pearson's chi-squared test and Fisher exact test were used for categorical variables, which were summarized as counts and percentages. For comparing proportions between groups, a 2-proportion *Z*-test was applied, with the alternative hypothesis assuming a difference between the proportions. Normality was assessed using the Shapiro-Wilk normality test; when the data significantly deviated from a normal distribution; the Wilcoxon-Mann-Whitney *U* test was used for continuous variables [15]. These continuous variables were expressed as medians with interquartile ranges (IQR). A *P*-value <.05 was considered statistically significant for all analyses.

Spatial analyses were conducted to examine possible relationships between socioeconomic indicators in the city's districts and the COVID-19 reinfection rate in each district. The Moran I statistic was employed to perform this analysis. The Global Indicator of Spatial Association was calculated to evaluate the overall spatial correlation between the actual and mean values of the reinfection rate, and the results were visualized using a Moran scatterplot. Moran I values range from −1 to +1, indicating negative or positive spatial autocorrelation, respectively.

Additionally, local indicators of spatial association (LISA) were used to identify local spatial clusters, or “hot spots” [16]. The LISA results are presented in a map with 5 possible classifications: High-High, High-Low, Low-High, Low-Low, and Not Significant (*P* > .05). High-High and Low-Low areas indicate that a region is surrounded by neighbors with similar values, whereas High-Low and Low-High classifications show that a region is surrounded by neighbors with contrasting values. For spatial weighting, a first-order queen contiguity structure was chosen.

Brazil's national currency “reais (R$)” was converted to US dollars (USD) using the mean value of the currency in the period of analysis, with data extracted from “Banco Central do Brasil” (Central Bank of Brazil), resulting in a value of US$1 = R$5.2 [17]. Data were manipulated in Knime Analytics Software (4.4.1 version), all statistical analyses were performed in R (4.2.0 version) programming language, and spatial analyses were elaborated in GeoDa free and open-source software (1.22.0 version) [18–20].

## RESULTS

**Figure 2.**
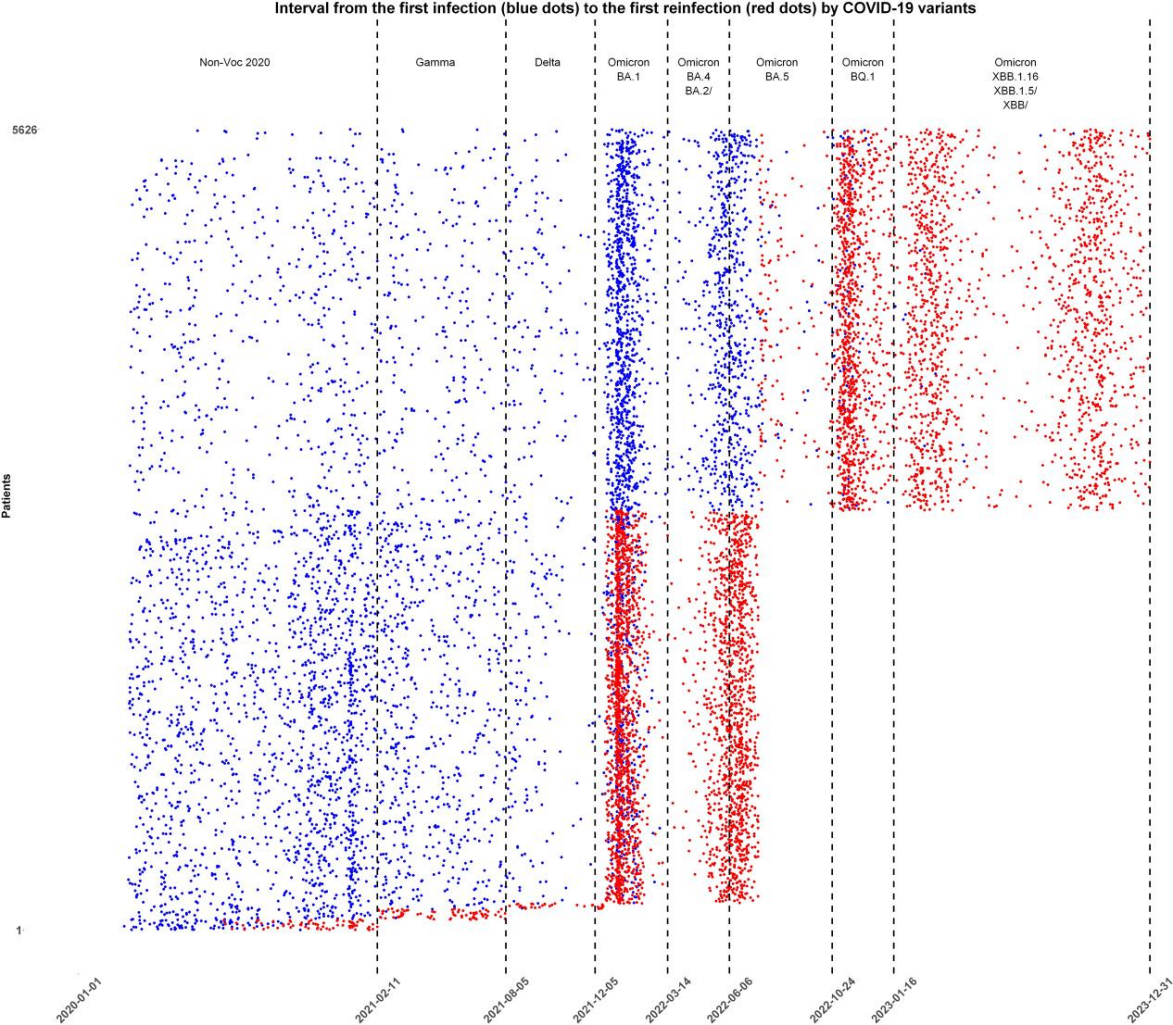
Interval graph showing the interval from the first infection to the first reinfection by COVID-19 variants. Each pair of dots represents 1 patient who experienced a reinfection, with blue dots marking initial infections and red dots marking reinfections. The timeline illustrates the first infection and reinfection events for each of the 5626 reinfected patients, with dates. Dashed black lines along the X-axis indicate the timing and the variants or subvariants associated with these events.

**Figure 3.**
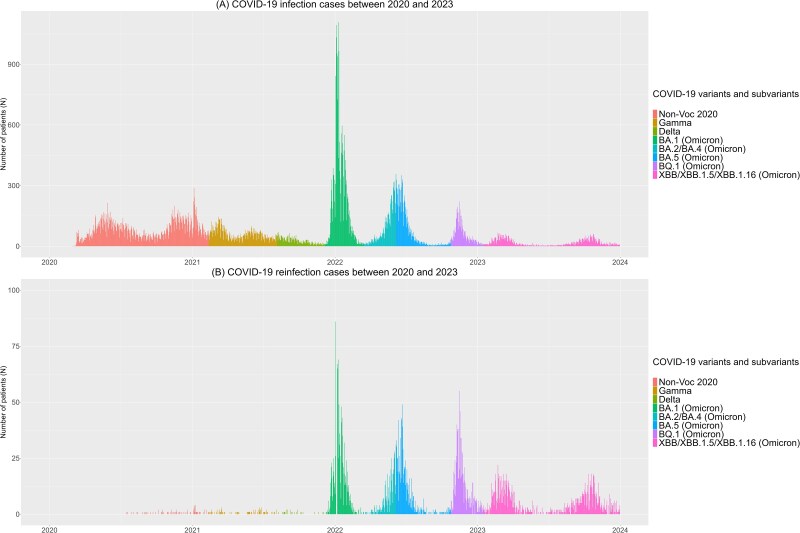
*A*, COVID-19 infection cases between 2020 and 2023 and (*B*) COVID-19 reinfection between 2020 and 2023.

Between 24 February 2020 and 31 December 2023, a total of 73 741 eligible patients had at least 1 positive COVID-19 test, with 5626 experiencing reinfection. Of these, 5344 (95.0%) had 1 reinfection, whereas 282 (5.0%) had 2 or more, including 270 with 2 reinfections, 11 with 3, and 1 with 4. The overall reinfection rate was 7.6 per 100 patients. Reinfections were rare during the pre-Omicron period, with a total of 174 cases occurring: 73 during the Non-Voc 2020 period, 76 during the Gamma variant period, and 25 during the Delta period. However, reinfections surged during the Omicron era, with 5452 cases distributed across several subvariants: BA.1 (1510 cases), BA.2/BA.4 (478 cases), BA.5 (927 cases), BQ.1 (984 cases), and XBB/XBB.1.5/XBB.1.16 (1553 cases) [Figure 1]. The highest reinfection rates were observed in patients initially infected during the BA.2/BA.4 and BA.5 periods, who were reinfected with XBB subvariants (4.6 and 3.8 per 100 patients, respectively) [Figure 2, Supplementary Materials 2 and 3]. Among those reinfected with XBB subvariants, 58.3% had their initial infection in the pre-Omicron and BA.1 periods. Reinfections remained uncommon until the emergence of Omicron, with the subvariants BA.1 producing the most infections and the XBB the most reinfections [Figure 3]. A total of 92 137 positive tests were recorded, averaging 1.2 tests per patient, with a higher ratio of 1.4 tests per patient during the Non-Voc 2020 period, which accounted for 34.0% of all tests [Supplementary Material 3]. During the Omicron period, the ratio dropped to 1.2, with 22 259 tests (24.2%) occurring during the BA.1 subvariant wave [Supplementary Material 3]. Reinfection rates varied significantly by variant, from 0.3% during the Non-Voc 2020 period to 13.8% during the Omicron era, with the highest rate (37.2%) observed for the XBB/XBB.1.5/XBB.1.16 subvariant [Supplementary Material 4]. The median time between initial infection and reinfection was 414 days (IQR: 296–592.8). The shortest reinfection interval was observed in the pre-Omicron period (median, 186.5 days), while the longest was during the XBB period (497 days) [Supplementary Material 5].

### Demographics

**Figure 4.**
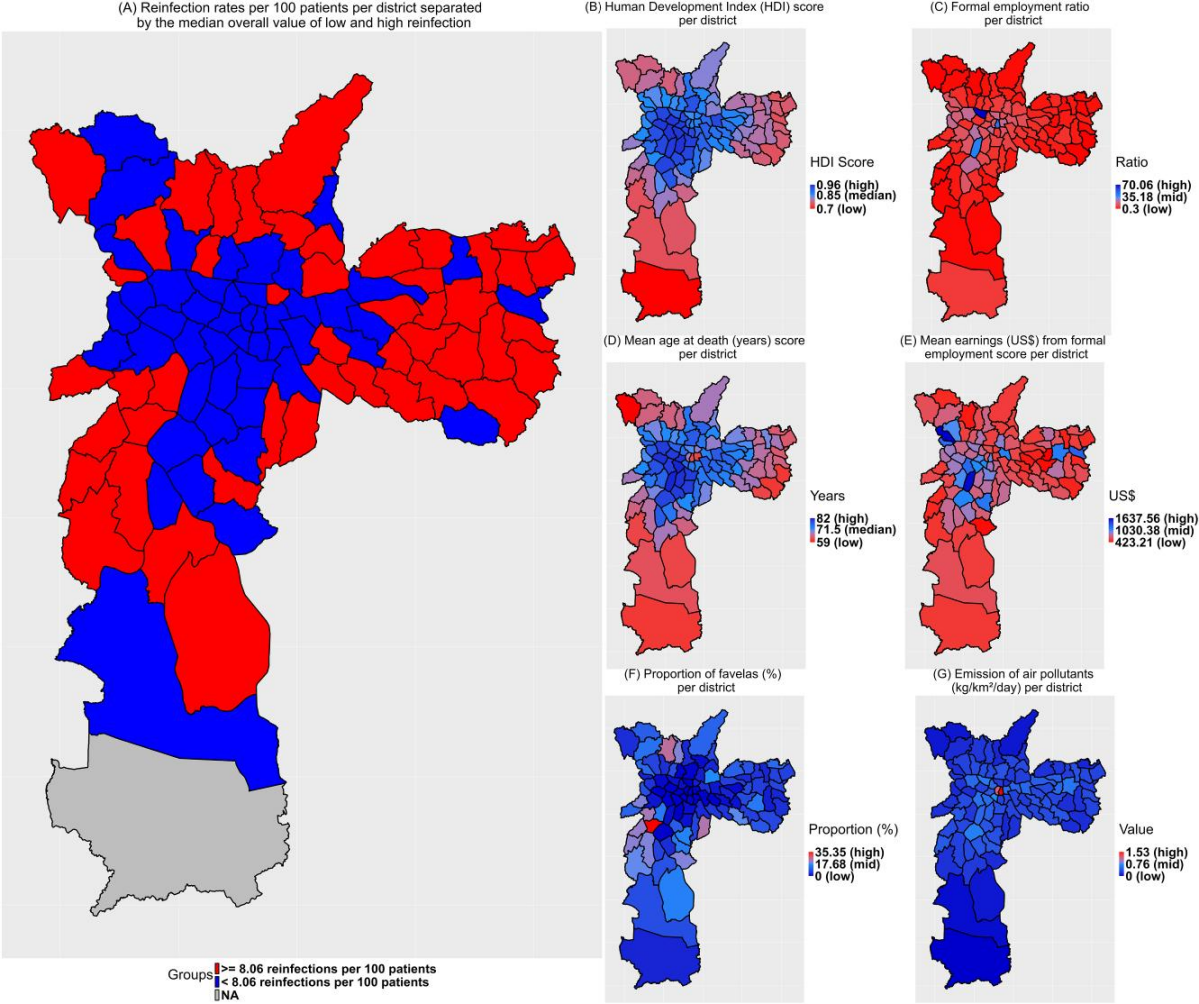
*A*, Reinfection rate per patient by district divided into 2 groups: “low reinfection rate” (values below the overall median of 8.06 reinfections per 100 patients) and “high reinfection rate” (values equal to or above this median); (*B*) Human Development Index (HDI) per district ranging from 0.70 (lowest value in the city) to 0.96 (highest value in the city); (*C*) formal employment ratio per district, ranging from 0.30 (low) to 70.06 (high); (*D*) mean age at death per district, ranging from 59 y (low) to 82 y (high); (*E*) mean earnings from formal employment per district, ranging from US$423.21 (low) to US$1637.56 (high); (*F*) proportion of favelas (slums) per district ranging from 0 (low) to 35.35 (high); and (*G*) emission of air pollutants (kg/km^2^/day) per district ranging from 0 (low) to 1.53 (high). The blue color represents better socioeconomic and lower reinfection rates, whereas red represents worse conditions. NA value (N = 1 district), shown in gray, indicates that no reinfections were observed in that district (missing value).

Reinfection rates per 100 patients were significantly higher among women than men (8.5 vs 6.6, *P* < .001) and older age groups (≥40 years) (7.8 vs 7.4, *P* = .022). Geographically, the highest reinfection rates were in the East region (9.6 per 100 patients), with notable differences across regions [Table 1]. Districts with higher reinfection rates were clustered in the North, South, and East, whereas lower rates were concentrated in the Central and West regions. The median reinfection rate across all 96 districts was 8.06 per 100 patients (IQR: 3.77). When dividing districts into “low” and “high” reinfection rate categories, it was evident that districts in the West, Central, and part of the South Zone had the lowest rates, whereas the majority of districts in the North, South, and East had the highest [Figure 4*A*]. By region, the highest median reinfection rate was in the East (9.72 per 100 patients, IQR: 4.83), followed by the North (8.48, IQR: 3.21), West (7.79, IQR: 1.44), South (7.65, IQR: 4.14), and Central (6.54, IQR: 1.93) [Table 2].

**Table 1.** Comparison of Groups of COVID-19–reinfected Patients and Non-reinfected Patients From 2020 to 2023 by Admission Characteristics

	Overall N = 73 741	Reinfected N = 5626	Non-reinfected N = 68 115	*P* Value
Year of reinfection, n (%)				<.001*
2020	17 198 (100.0%)	45 (0.3%)	17 153 (99.7%)	
2021	16 700 (100.0%)	329 (2.0%)	16 371 (98.0%)	
2022	33 942 (100.0%)	3652 (10.8%)	30 290 (89.2%)	
2023	5901 (100.0%)	1600 (27.1%)	4301 (72.9%)	
Sex, n (%)				<.001*
Female	39 116 (100.0%)	3344 (8.5%)	35 772 (91.5%)	
Male	34 506 (100.0%)	2282 (6.6%)	32 224 (93.4%)	
Unknown	119	0	119	
Age, median (P25-P75)	41.0 (29.0–52.0)	41.0 (31.0–51.0)	41.0 (29.0–52.0)	<.001**
Age group, n (%)				<.001***
≤17 y	9103 (100.0%)	333 (3.7%)	8770 (96.3%)	
18–39 y	24 294 (100.0%)	2129 (8.8%)	22 165 (91.2%)	
40–59 y	28 833 (100.0%)	2403 (8.3%)	26 430 (91.7%)	
>60 y	11 511 (100.0%)	761 (6.6%)	10 750 (93.4%)	
Nationality, n (%)				<.001****
Brazilian	70 336 (100.0%)	5499 (7.8%)	64 837 (92.2%)	
Foreign	3142 (100.0%)	124 (3.9%)	3018 (96.1%)	
Unknown	263	3	260	
City regions, n (%)				<.001***
East	4985 (100.0%)	478 (9.6%)	4507 (90.4%)	
North	4484 (100.0%)	350 (7.8%)	4134 (92.2%)	
South	30 923 (100.0%)	2388 (7.7%)	28 535 (92.3%)	
West	29 519 (100.0%)	2165 (7.3%)	27 354 (92.7%)	
Central	3830 (100.0%)	245 (6.4%)	3585 (93.6%)	

^*^Pearson's chi-squared test.

^**^Wilcoxon rank-sum test.

^***^Fisher exact test for count data with simulated *P* value (based on 2000 replicates).

^****^Fisher exact test for count data.

**Table 2.** Comparison of the Districts With Reinfections by Inequality Indicators Divided by Groups of Low (Below Median) Reinfection Rates and High (Equal or Above Median) Reinfection Rates

Reinfection Rate Groups (Low vs. High)		HDI, Median (P25-P75)	Formal Employment Ratio, Median (P25-P75)	Mean Age at Death (y), Median (P25-P75)	Proportion of Favelas (%), Median (P25-P75)	Mean Earnings (US$), Median (P25-P75)	Emission of Air Pollutants (kg/km^2^/day), Median (P25-P75)
Central	*P* value	.095*	.30*	.095*	.91*	.40*	.21*
	Overall, N = 10	0.90 (0.86–0.93)	13.55 (10.65–18.86)	72.00 (68.25–74.75)	0.00 (0.00–0.26)	745.1 (623.9–944.4)	0.47 (0.29–0.69)
	<6.54**, N = 5	0.87 (0.86–0.90)	18.28 (14.87–19.05)	68.00 (63.00–71.00)	0.00 (0.00–0.18)	676.6 (606.4–749.2)	0.55 (0.52–1.16)
	≥ 6.54**, N = 5	0.94 (0.90–0.94)	10.82 (10.59–12.24)	74.00 (73.00–76.00)	0.00 (0.00–0.28)	880.1 (741.0–995.2)	0.30 (0.29–0.41)
	*P* value	.037*	.043*	.012*	.024*	.073*	.073*
West	Overall, N = 15	0.93 (0.88–0.95)	12.91 (4.20–19.66)	77.00 (75.00–80.00)	1.31 (0.30–9.47)	883.4 (745.1–1024.8)	0.27 (0.20–0.52)
	<7.79**, N = 7	0.95 (0.93–0.96)	19.85 (14.33–31.06)	80.00 (78.50–81.50)	0.51 (0.04–0.90)	904.0 (890.9–1136.2)	0.52 (0.36–0.55)
	≥ 7.79**, N = 8	0.88 (0.85–0.93)	8.43 (3.57–12.91)	75.00 (71.75–76.50)	9.47 (2.32–15.88)	793.6 (579.6–908.8)	0.22 (0.19–0.29)
South	*P* value	.003*	.001*	.003*	<.001*	.003*	.091*
	Overall, N = 21	0.84 (0.80–0.92)	2.06 (1.02–7.92)	70.00 (67.00–77.00)	9.97 (3.69–20.26)	692.2 (547.2–802.2)	0.31 (0.18–0.39)
	<7.65**, N = 10	0.93 (0.90–0.94)	7.98 (4.29–9.09)	78.00 (74.00–80.00)	3.46 (0.86–7.50)	808.7 (719.7–940.7)	0.36 (0.30–0.40)
	≥ 7.65**, N = 11	0.81 (0.78–0.83)	1.17 (0.80–1.84)	67.00 (65.00–69.00)	20.26 (14.90–21.10)	547.2 (496.3–682.8)	0.23 (0.17–0.32)
North	*P* value	.79*	.66*	.89*	.29*	.60*	.60*
	Overall, N = 18	0.84 (0.81–0.86)	2.29 (0.90–4.59)	70.50 (66.50–73.00)	6.96 (2.99–11.75)	598.4 (544.2–730.5)	0.24 (0.16–0.31)
	<8.48**, N = 9	0.85 (0.79–0.87)	2.58 (0.84–6.86)	72.00 (65.00–73.00)	4.02 (2.06–10.29)	607.4 (550.2–831.9)	0.19 (0.15–0.31)
	≥ 8.48**, N = 9	0.84 (0.82–0.85)	2.09 (1.07–3.93)	70.00 (68.00–72.00)	8.21 (3.04–17.20)	589.5 (535.8–653.6)	0.26 (0.21–0.30)
East	*P* value	.027*	.031*	.031*	.060*	.74*	.92*
	Overall, N = 31	0.80 (0.77–0.86)	1.92 (1.03–3.67)	68.00 (66.00–73.00)	5.21 (2.69–7.14)	576.2 (480.2–631.3)	0.31 (0.25–0.37)
	<9.72**, N = 15	0.87 (0.79–0.89)	3.09 (1.73–4.62)	73.00 (67.00–75.50)	4.37 (1.26–6.12)	576.2 (530.8–622.2)	0.31 (0.25–0.39)
	≥ 9.72**, N = 16	0.80 (0.77–0.81)	1.21 (0.85–2.04)	68.00 (65.75–70.00)	6.26 (3.27–9.05)	567.1 (467.6–667.3)	0.31 (0.20–0.35)
All regions	*P* value	<.001*	<.001*	<.001*	<.001*	<.001*	.019*
	Overall, N = 95	0.85 (0.80–0.90)	3.70 (1.26–9.70)	72.00 (67.00–76.00)	4.38 (0.91–10.49)	652.3 (540.2–830.1)	0.30 (0.20–0.40)
	<8.06**, N = 48	0.90 (0.85–0.94)	8.04 (3.99–13.69)	74.00 (71.00–78.25)	1.18 (0.15–6.36)	758.2 (598.5–927.3)	0.32 (0.23–0.49)
	≥ 8.06**, N = 47	0.82 (0.79–0.85)	1.67 (0.93–3.20)	68.00 (66.00–72.00)	7.30 (3.54–14.90)	580.4 (525.1–666.8)	0.28 (0.18–0.33)

*Wilcoxon test with no correction.

**Reinfections per 100 patients.

### Socioeconomic Indicators and Spatial Correlations

Regions with low and high reinfection rates demonstrated statistically significant differences in socioeconomic indicators. The HDI was higher in regions with lower reinfection rates, with a median of 0.90 compared to 0.82 in high-reinfection areas (*P* < .001). This trend held across the East (0.87 vs 0.80, *P* = .027) and South (0.93 vs 0.81, *P* = .003) and was reflected in mean age at death and formal employment rates, both higher in low-reinfection regions. Significant disparities were observed in the proportion of favelas, particularly in the South (3.5% vs 20.3%, *P* < .001) and West (0.5% vs 9.5%, *P* = .024), while mean earnings also differed markedly across all regions (US$758.2 vs US$580.4, *P* < .001), with the South showing the largest difference (US$808.7 vs US$547.2, *P* = .003). Air pollutant emissions were slightly higher in high-reinfection regions (0.28 vs 0.30 kg/km^2^/day, *P* = .019) [Table 2].

Mapping the geographic distribution of variables associated with reinfection rates showed HDI values generally high across the city, with lower values in peripheral areas [Figure 4*B*]. The highest values for mean age at death, formal employment, and mean earnings were concentrated in central and western districts [Figure 4*C*, *D*, *E*]. The proportion of favelas and air pollutant emissions, although generally low, were more pronounced in central districts [Figure 4*F*, *G*].

**Figure 5.**
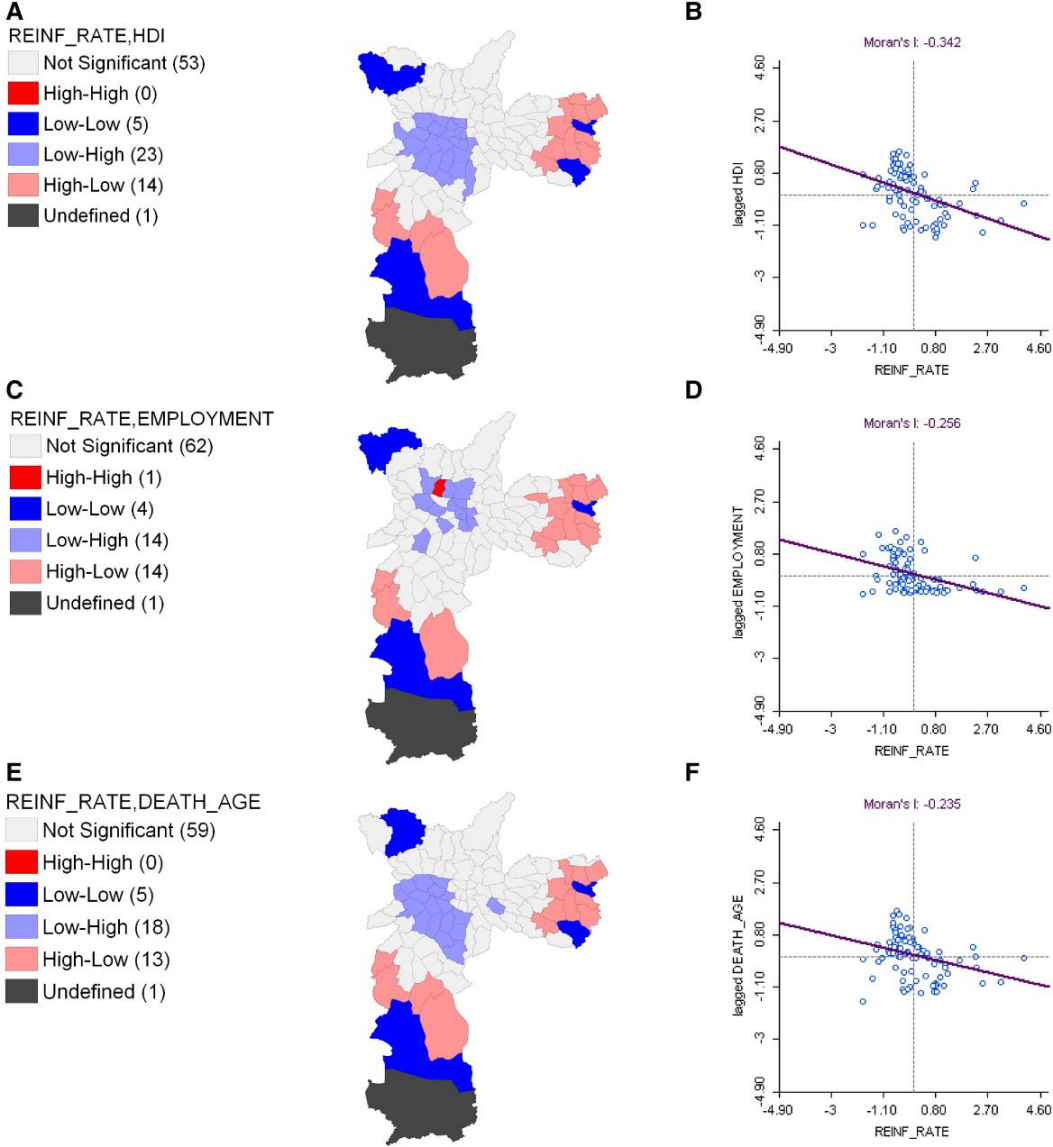

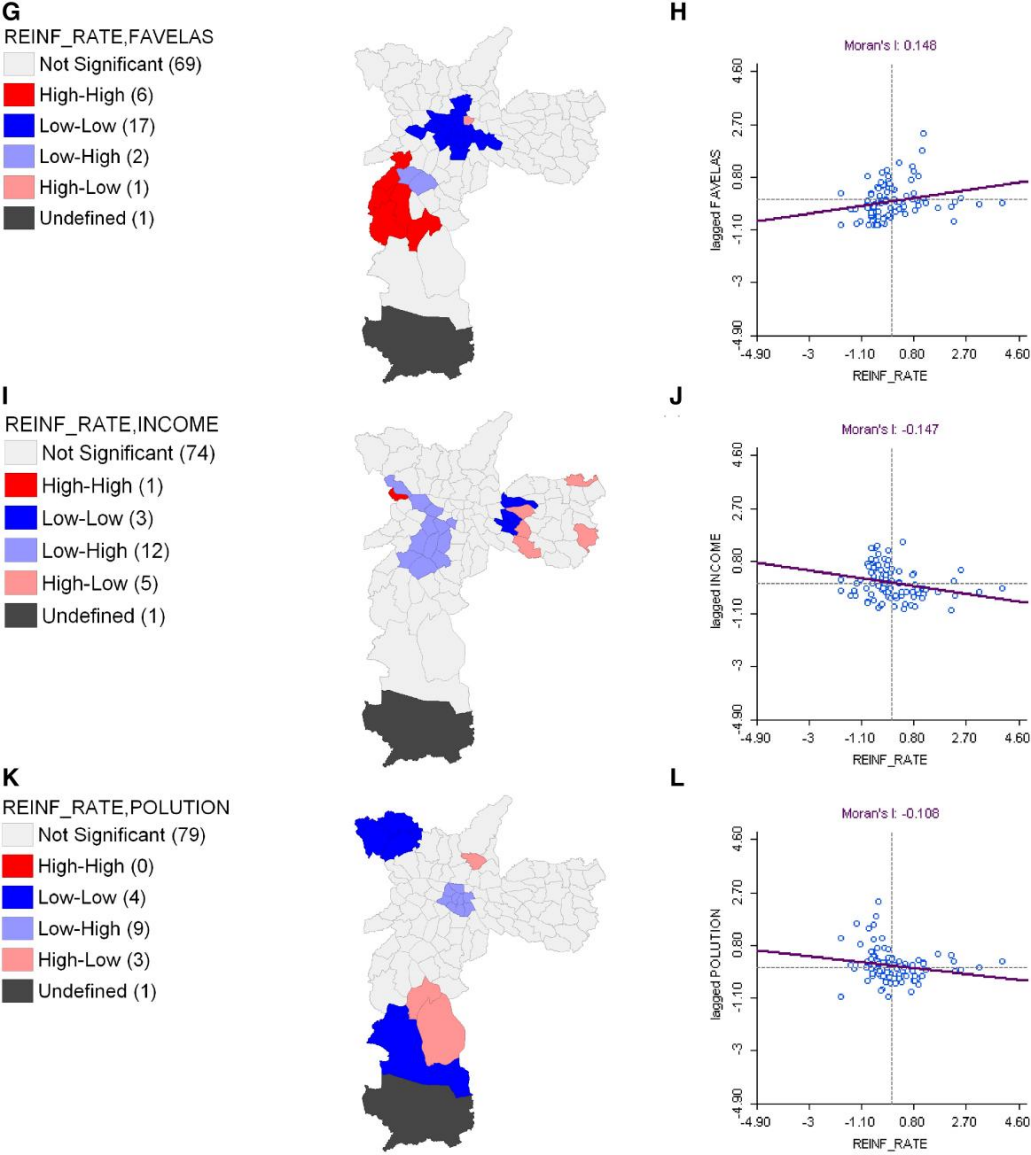
Bivariate local indicators of spatial associations of socioeconomic variables: HDI *(A)*, formal employment *(C)*, mean age at death *(E)*, proportion of favelas *(G)*, mean income *(I)*, and pollution *(K)*. Additionally, a Moran scatterplot with the global spatial association indicator value *(B, D, F, H, J, L)* is provided for the respective variables and the reinfection rate per 100 patients per district. The figures on the left display the LISA classifications with 4 possible outcomes: “not significant” (*P* > .05), “High-High,” “Low-High,” “High-Low,” “Low-Low,” with the number of districts in each category shown in parentheses. On the right is the Moran scatterplot for the reinfection rate per 100 patients per district alongside each pair of socioeconomic indicators.

The strongest spatial correlation with reinfection rates was seen with HDI (Moran I = −0.342), indicating that low-reinfection districts clustered near areas with high HDI, particularly in the central region, where 23 districts were classified as “Low-High” [Figure 5*A*, *B*]. In contrast, 14 districts in the East and South displayed both high reinfection rates and low HDI values [Figure 5*A*]. The formal employment rate also showed a similar spatial correlation (Moran I = −0.256), with high-reinfection, low-employment districts in the East and far South, whereas the Central and North regions contained low-reinfection, high-employment districts; 1 district in the North showed both high reinfection rates and high employment [Figure 5*C*].

Other indicators, such as mean age at death, followed a similar spatial trend with a weaker correlation (Moran I = −0.235) [Figure 5*E*, *F*]. The proportion of favelas was the only indicator with a positive spatial correlation (Moran I = +0.148), indicating clustering of high-reinfection districts with high-favela areas, observed in 17 districts in the Central and 6 in the South [Figure 5*G*, *H*]. Reinfection rates were lowest in districts surrounded by areas with high mean income, especially in 12 central districts (Moran I = −0.147) [Figure 5*I*, *J*]. Although pollution had a low correlation with reinfection rates (Moran I = −0.108), some central districts exhibited low reinfection rates despite high pollution levels [Figure 5*K*, *L*].

## DISCUSSION

This study demonstrates the critical role of socioeconomic and demographic factors in shaping COVID-19 reinfection patterns. Reinfection was more common among females, younger individuals, and those living in socioeconomically disadvantaged regions of São Paulo, particularly in areas with lower HDI and higher proportions of favelas. Addressing social vulnerabilities and inequalities in public health strategies is essential to mitigating reinfection risks. The emergence of the Omicron variant, with its immune evasion capabilities, highlights the importance of continuous genomic surveillance and targeted interventions to manage reinfection across diverse populations.

Several key demographic differences were observed between individuals who experienced reinfection and those who did not. A notable finding was the higher proportion of females experiencing reinfection compared to males [21, 22]. This gender disparity in reinfection rates is statistically significant and warrants further investigation into possible behavioral [23], or sociocultural factors that may contribute to this difference. For instance, women are generally more likely to seek healthcare or get tested compared to men, which could contribute to the greater detection of reinfections in women [23]. Furthermore, gender differences in healthcare utilization patterns have been well-documented, with women often having higher rates of healthcare visits and interaction with health professionals, potentially resulting in more frequent diagnoses and subsequent reinfections being reported [24]. Although our findings are statistically significant, the clinical relevance of the 1.9 per 100 patients’ difference in reinfection rates between genders may be modest and should be interpreted with caution as such differences may reflect statistical variation rather than meaningful biological differences. Age also played a role, with reinfected individuals being slightly younger. Although the age distribution was similar, younger individuals may have higher exposure levels or distinct immune responses influencing reinfection risk [25].

Geographically, higher reinfection rates were concentrated in the East and the North regions of Sao Paulo, which likely reflects specific socioeconomic and demographic vulnerabilities. Reinfection was more common in districts with lower HDI, fewer formal employment opportunities, and higher proportions of favelas (informal settlements). These findings illustrate the link between social vulnerability and health outcomes [3]. Specifically, socioeconomically disadvantaged areas appear to be more exposed to COVID-19 and face greater barriers to effective mitigations measures, including access to healthcare and preventive interventions. In São Paulo, these interventions included widespread vaccination campaigns, mass testing, social assistance programs, and telemedicine services, which may have been less accessible in underserved communities.

We found significant spatial correlations between socioeconomic factors and reinfection rates, especially in low-HDI regions. Districts with high reinfection rates often bordered others with low HDI, forming clusters of vulnerability. Indicators such as employment rates, income, and the proportion of favelas were key, with the latter showing the only positive spatial correlation with reinfection. This points to the elevated risk in densely populated, low-income areas where preventive measures may be harder to implement. Air pollution levels were also higher in central districts, though its correlation with reinfection was weaker than other factors. Pollution remains relevant as it can worsen respiratory conditions, particularly in areas already facing high reinfection rates.

The emergence of the Omicron variant, characterized by a multitude of mutations, has significantly impacted the landscape of COVID-19 reinfection [26]. The Omicron variant, particularly its subvariants BA.1, BA.5, and XBB, played a pivotal role in the sharp rise in reinfection rates [26]. The transmissibility and immune evasion capabilities of Omicron, as evidenced by the high reinfection rates in the Omicron period (13.8%), reflect its capacity to circumvent both natural immunity and vaccine-induced protection [1]. The high reinfection rates, especially in XBB (37.2%), highlight the need for ongoing genomic surveillance and adaptation of public health strategies to effectively manage emerging variants.

This study has several limitations that may impact the interpretation of the results. The reliance on retrospective data from multiple testing sites introduces potential inconsistencies in data collection and reporting practices. Variations in testing availability, diagnostic criteria, and report accuracy across different periods and locations could influence the observed reinfection rates. Additionally, the study is limited to a specific geographic region (São Paulo), and a select group of diagnostic facilities, which may not fully represent the broader population or account for regional differences in COVID-19 exposure and health care access. Another significant limitation is the lack of detailed clinical data and information on vaccination status, which could provide critical context for understanding reinfection dynamics. Because of the sensitive nature of personal information within vaccination data, access to this information in national databases is limited under the General Data Protection Law established in 2020 in Brazil. Consequently, these data were not included in the analyses. However, São Paulo's COVID-19 vaccination campaign followed a phased rollout, with initial doses prioritized for healthcare workers and older adults in early 2021, expanding to the general adult population later that year, followed by booster campaigns from late 2021 onward. From 2021 to 2023, São Paulo achieved >90% vaccination coverage among adults for COVID-19 with at least 1 dose [27]. Furthermore, the socioeconomic and demographic data, while insightful, were incomplete for a substantial portion of the population, particularly concerning ethnicity and income. Additionally, other potential factors associated with reinfection rates, such as comorbidities and immunosuppression, were not analyzed, and adjustments for multiple confounding variables were not performed. Because all tests were performed exclusively at HIAE, cases in which individuals sought testing at different laboratories or hospitals, as well as asymptomatic or mild reinfections that did not prompt medical attention, were not captured in our analysis. Furthermore, the availability and use of home testing during the study period could also have contributed to underestimating the true reinfection rate. The variants analyzed in this study were classified based on the predominant variant (≥75% of sequenced virus) reported weekly by the Global Initiative on Sharing Avian Influenza Data for São Paulo, Brazil, as only a small proportion of positive samples were sequenced [14]. Last, although the study uses regional socioeconomic factors, there is a risk of ecological fallacy because individuals living in the same neighborhood may not share the same HDI or other socioeconomic factors, potentially affecting the interpretation of the data at the individual level.

Future studies should aim to address these gaps by incorporating more detailed and comprehensive data to enhance the understanding of COVID-19 reinfection patterns and their underlying causes.

In conclusion, this study demonstrates the important role of socioeconomic factors in COVID-19 reinfection patterns. Addressing social vulnerabilities, especially in low-income and densely populated areas, should be a key focus of public health strategies. Interventions need to prioritize improving healthcare access, vaccination, and preventive education in disadvantaged regions. By identifying the prevalence of reinfection and key predictors, such as age, gender, and socioeconomic factors, this research offers valuable insights into reinfection dynamics within diverse socioeconomic contexts, particularly in a middle-income country. As new variants emerge, continued surveillance and targeted interventions will be vital to reducing reinfections and protecting vulnerable populations.

## Supplementary Material

ofaf181_Supplementary_Data

## Supplementary Data

Supplementary materials are available at *Open Forum Infectious Diseases* online. Consisting of data provided by the authors to benefit the reader, the posted materials are not copyedited and are the sole responsibility of the authors, so questions or comments should be addressed to the corresponding author.

## Notes

***Acknowledgments.*** The authors express gratitude to all of the health professionals at Hospital Israelita Albert Einstein who were willing to donate their time to save lives.

***Author Contributions.*** All authors contributed substantially to the manuscript and agreed to the final submitted version.

***Data sharing agreement.*** Deidentified individual study data reported in this article can be requested upon application to the investigator board via the corresponding author.

***Financial support.*** No funding was received related to this article.

***Potential conflicts of interest**.* All authors: No reported conflicts.
