# Supplementary material for: COVID-19 Reinfections in the City of São Paulo, Brazil: Prevalence and Socioeconomic Factors
Source: Open Forum Infect Dis. 2025 Apr 16;12(4):ofaf181. doi: 10.1093/ofid/ofaf181 (PMC12000647; doi:10.1093/ofid/ofaf181)
Supplement: ofaf181_Supplementary_Data [file ofaf181_supplementary_data.docx]

**COVID-19 Reinfections in**

**the City of São Paulo, Brazil: Prevalence and Socioeconomic Factors**

Supplemental Material

Daniel Tavares Malheiro, BSc^1^; Kaue Capellato Junqueira Parreira, BSc^1^; Patricia Deffune Celeghini, MD^1^; Gustavo Yano Callado^1^; André Luis Franco Cotia, MD^1^; Miguel Cendoroglo Neto, MD^1^; Marcelo A. S. Bragatte, PhD^2,3^; Isaac Negretto Schrarstzhaupt^2,3,4^; Vanderson Sampaio, PhD^2^; Takaaki Kobayashi, MD^5,6^; Michael B. Edmond, MD^7^; Alexandre R. Marra, MD^1,6^

1. Hospital Israelita Albert Einstein, São Paulo, Brazil
2. Instituto Todos Pela Saúde, São Paulo, Brazil
3. Instituto Capixaba de Ensino, Pesquisa e Inovação em Saúde ICEPi, Espírito Santo, Brazil
4. Faculdade de Saúde Pública, Universidade de São Paulo, São Paulo, Brazil
5. Department of Internal Medicine, University of Kentucky, Lexington, KY, USA
6. Department of Internal Medicine, University of Iowa, Iowa City, IA, USA
7. West Virginia University School of Medicine, Morgantown, WV, USA

1. Contents

1. Cover Page…………………………………………………………………………………1
2. Contents……………………………………………………………………………………2
3. Supplementary Material 1…………………………………………………………………3
4. Supplementary Material 2……………………………………………………………......4
5. Supplementary Material 3…………………………………………………………………5
6. Supplementary Material 4……………………………………………………………......6
7. Supplementary Material 5…………………………………………………………………7

**Supplementary Material 1**: Names and descriptions of selected variables considered in the analysis of the indicators for districts in São Paulo

| **Formal employment ratio** | Absolute number of formal jobs at the reference date divided by the active age population (aged >15 years) |
| --- | --- |
| **Mean age at death** | Sum of ages at death divided by total deaths by all ages * 100 |
| **Proportion of slums (“favelas” in Portuguese)** | Estimated number of slums divided by total number of permanent private households |
| **Mean earnings** | Nominal wage mass (US$ at the reference date divided by the absolute number of formal jobs at the reference date) |
| **Emission of air pollutants** | District’s pollution emission (kg/year) divided by the district’s area (km²) |

**Supplementary Material 2:** Cross table of infected and reinfected patients by the dominance of COVID-19 variants and subvariants. For reinfected patients, each percentage can also be interpreted as the reinfection rate per 100 patients.

| **COVID-19 Infected**  **patients** | | | **COVID-19 Reinfected patients** | | | | | | | |
| --- | --- | --- | --- | --- | --- | --- | --- | --- | --- | --- |
|  |  |  | **Pre Omicron** | **Omicron** | | | | | **Without reinfection** | Total |
|  |  |  |  | **BA.1** | **BA.2/**  **BA.4** | **BA.5** | **BQ.1** | **XBB/**  **XBB.1.5/**  **XBB.1.16** |  |  |
| **Pre Omicron** | | N | 174 | 1,510 | 375 | 562 | 376 | 452 | 30,765 | 34,214 |
|  |  | Row % | 0.5% | 4.4% | 1.1% | 1.6% | 1.1% | 1.3% | 89.9% |  |
| **Omicron** | **BA.1** | N |  | 0 | 103 | 358 | 480 | 453 | 17,365 | 18,759 |
|  |  | Row % |  | 0% | 0.5% | 1.9% | 2.6% | 2.4% | 92.6% |  |
|  | **BA.2/**  **BA.4** | N |  |  | 0 | 2 | 74 | 286 | 5,856 | 6,218 |
|  |  | Row % |  |  | 0% | 0.0% | 1.2% | 4.6% | 94.2% |  |
|  | **BA.5** | N |  |  |  | 5 | 54 | 261 | 6,606 | 6,926 |
|  |  | Row % |  |  |  | 0.1% | 0.8% | 3.8% | 95.4% |  |
|  | **BQ.1** | N |  |  |  |  | 0 | 86 | 3,366 | 3,452 |
|  |  | Row % |  |  |  |  | 0% | 2.5% | 97.5% |  |
|  | **XBB/**  **XBB.1.5/**  **XBB.1.16** | N |  |  |  |  |  | 15 | 4,157 | 4,172 |
|  |  | Row % |  |  |  |  |  | 0.4% | 99.6% |  |
| Total | | N | 174 | 1,510 | 478 | 927 | 984 | 1,553 | 68,115 | 73,741 |

**Supplementary Material 3:** Descriptive numbers of positive COVID-19 tests, tested patients, reinfections and reinfection rates included in the study.

| **COVID-19 Variants** | | **Positive tests**  **(%)** | **Patients**  **(%)** | **Positive tests/**  **Patients** | **Reinfections**  **(%)** | **Reinfection rate**  **per 100 tests** | **Reinfection rate**  **per 100 patients** |
| --- | --- | --- | --- | --- | --- | --- | --- |
| **Pre Omicron** | Non-Voc 2020 | 31,370  (34.0) | 22,652  (30.7) | 1.4 | 73  (1.3) | 0.2 | 0.3 |
|  | Gamma | 11,181  (12.1) | 8,859  (12.0) | 1.3 | 76  (1.4) | 0.7 | 0.9 |
|  | Delta | 3,428  (3.7) | 2,703  (3.7) | 1.3 | 25  (0.4) | 0.7 | 0.9 |
| **Omicron** | BA.1 | 22,259  (24.2) | 18,759  (25.4) | 1.2 | 1,510  (26.8) | 6.8 | 8.0 |
|  | BA.2/  BA.4 | 7,429  (8.1) | 6,218  (8.4) | 1.2 | 478  (8.5) | 6.4 | 7.7 |
|  | BA.5 | 8,119  (8.8) | 6,926  (9.4) | 1.2 | 927  (16.5) | 11.4 | 13.4 |
|  | BQ.1 | 3,901  (4.2) | 3,452  (4.7) | 1.1 | 984  (17.5) | 25.2 | 28.5 |
|  | XBB/  XBB.1.5/  XBB.1.16 | 4,450  (4.8) | 4,172  (5.7) | 1.1 | 1,553  (27.6) | 34.9 | 37.2 |
| **Total** | | 92,137  (100) | 73,741  (100) | 1.2 | 5,626  (100) | 6.1 | 7.6 |


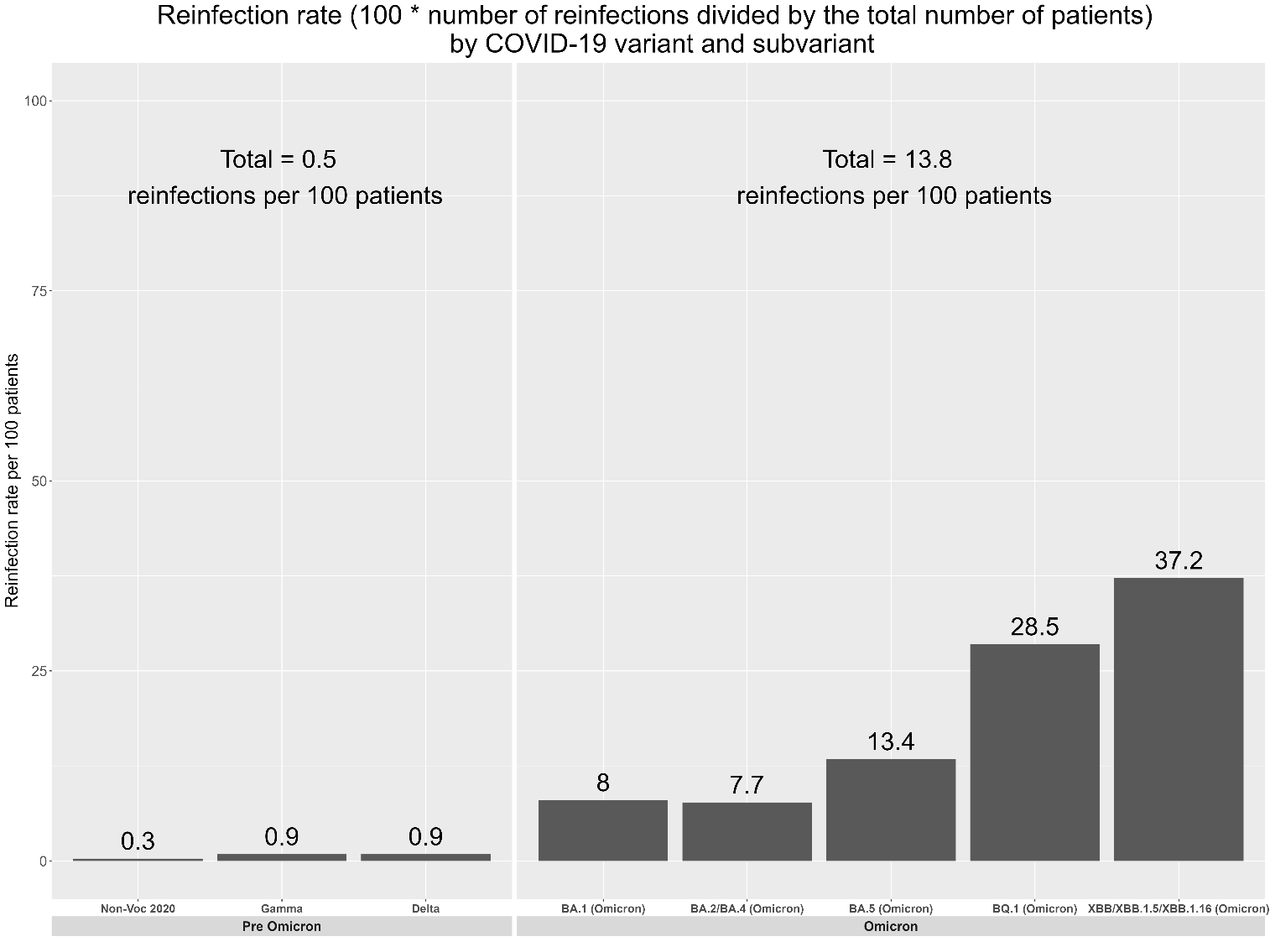
**Supplementary Material 4:** COVID-19 reinfection rates per 100 patients by variants and subvariants, separated into Pre-Omicron and Omicron periods.

**Alt text**: Bar plots showing the reinfection rates per 100 patients for each COVID-19 variant and subvariant, as well as the overall value by variant (Pre-Omicron and Omicron). The Pre-Omicron variant had a reinfection rate of 0.5 per 100 patients, while the Omicron variant had a rate of 13.8 per 100 patients, with the XBB subvariant showing the highest value of 37.2 reinfections per 100 patients.


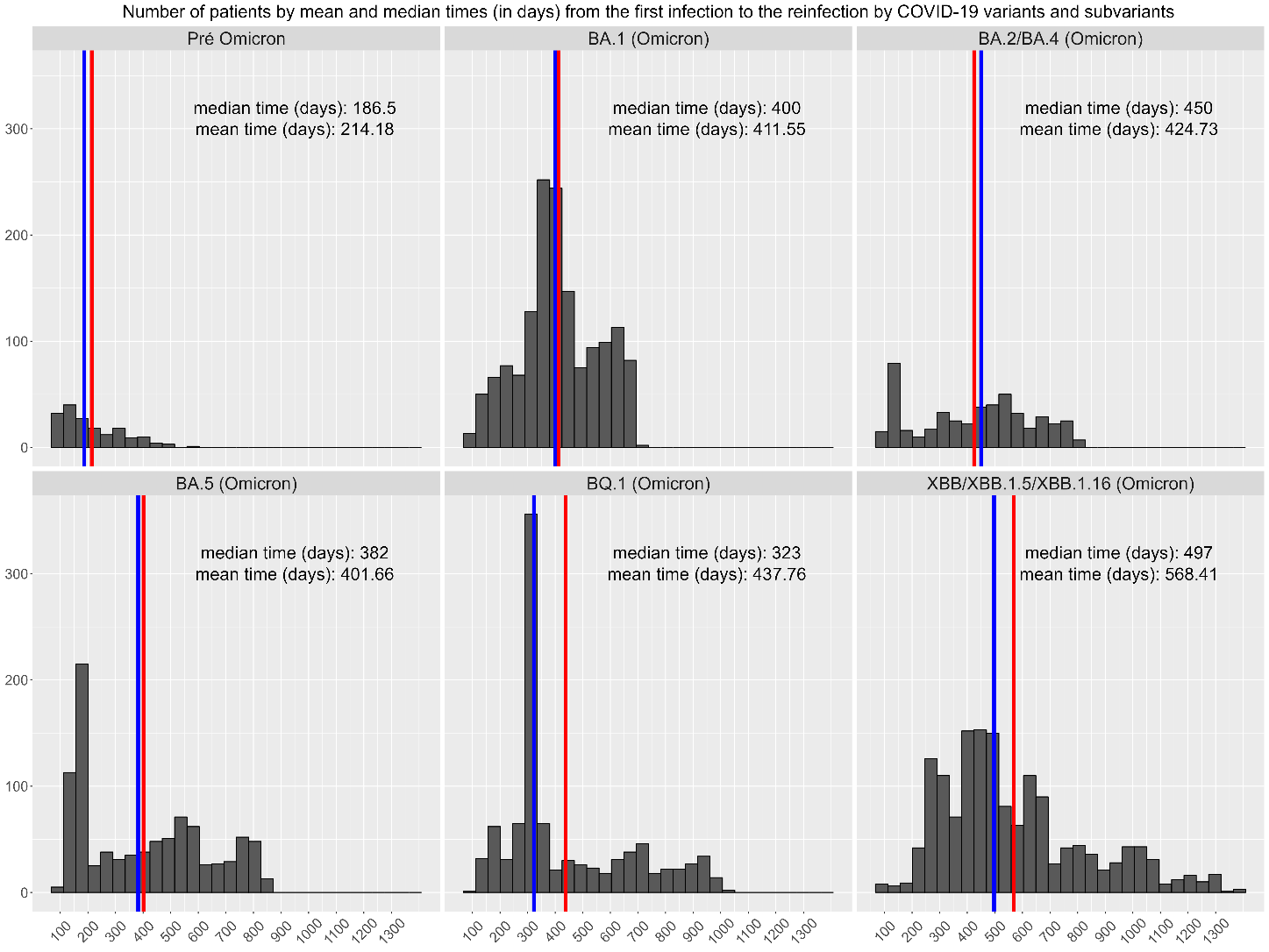
**Supplementary Material 5:** Time elapsed in days from the first infection to the first reinfection, categorized by COVID-19 variants and subvariants.

**Footnote**: The blue vertical line is the median and the red line is the mean of the period, for all patients, between the first infection and the first reinfection in days.

**Alt text**: Histograms showing the time (in days) between the infection and the first reinfection for each COVID-19 variant and subvariant, highlighting a substantial increase in this duration for the Omicron subvariants compared to the overall Pre-Omicron variants. The Pre-Omicron variants had the lowest overall median (186.5 days) and mean (214.2 days), while the XBB subvariant had the highest median (497.0 days) and mean (568.4 days).
